# Inflammation leads to distinct populations of extracellular vesicles from microglia

**DOI:** 10.1186/s12974-018-1204-7

**Published:** 2018-05-28

**Authors:** Yiyi Yang, Antonio Boza-Serrano, Christopher J. R. Dunning, Bettina Hjelm Clausen, Kate Lykke Lambertsen, Tomas Deierborg

**Affiliations:** 1 Department of Experimental Medical Science, Experimental Neuroinflammation Laboratory, Lund University, Lund, Sweden; 2 Department of Biochemistry and Structural Biology, Lund University, Lund, Sweden; 3 Department of Neurobiology Research, Institute of Molecular Medicine, University of Southern Denmark, Odense, Denmark; 4 BRIGDE—Brain Research–Inter-Disciplinary Guided Excellence, Department of Clinical Research, University of Southern Denmark, Odense, Denmark; 5 Department of Neurology, Odense University Hospital, Odense, Denmark

**Keywords:** Microglia, Extracellular vesicles (EVs), Neuroinflammation, TNF

## Abstract

**Background:**

Activated microglia play an essential role in inflammatory responses elicited in the central nervous system (CNS). Microglia-derived extracellular vesicles (EVs) are suggested to be involved in propagation of inflammatory signals and in the modulation of cell-to-cell communication. However, there is a lack of knowledge on the regulation of EVs and how this in turn facilitates the communication between cells in the brain. Here, we characterized microglial EVs under inflammatory conditions and investigated the effects of inflammation on the EV size, quantity, and protein content.

**Methods:**

We have utilized western blot, nanoparticle tracking analysis (NTA), and mass spectrometry to characterize EVs and examine the alterations of secreted EVs from a microglial cell line (BV2) following lipopolysaccharide (LPS) and tumor necrosis factor (TNF) inhibitor (etanercept) treatments, or either alone. The inflammatory responses were measured with multiplex cytokine ELISA and western blot. We also subjected TNF knockout mice to experimental stroke (permanent middle cerebral artery occlusion) and validated the effect of TNF inhibition on EV release.

**Results:**

Our analysis of EVs originating from activated BV2 microglia revealed a significant increase in the intravesicular levels of TNF and interleukin (IL)-6. We also observed that the number of EVs released was reduced both in vitro and in vivo when inflammation was inhibited via the TNF pathway. Finally, via mass spectrometry, we identified 49 unique proteins in EVs released from LPS-activated microglia compared to control EVs (58 proteins in EVs released from LPS-activated microglia and 37 from control EVs). According to Gene Ontology (GO) analysis, we found a large increase of proteins related to translation and transcription in EVs from LPS. Importantly, we showed a distinct profile of proteins found in EVs released from LPS treated cells compared to control.

**Conclusions:**

We demonstrate altered EV production in BV2 microglial cells and altered cytokine levels and protein composition carried by EVs in response to LPS challenge. Our findings provide new insights into the potential roles of EVs that could be related to the pathogenesis in neuroinflammatory diseases.

**Electronic supplementary material:**

The online version of this article (10.1186/s12974-018-1204-7) contains supplementary material, which is available to authorized users.

## Background

Microglia are considered the main innate-immune cells of the central nervous system (CNS). They continuously survey their microenvironment and have the ability to interact with neurons to regulate their activity [1]. In the healthy brain, microglia continuously survey their surroundings with highly dynamic processes [2] and become activated in response to injury, infection or neurodegenerative processes [3]. Emerging evidence has shown that microglia are key causative players in neuroinflammation, which in turn is believed to play a major role in neurodegenerative diseases [4].

Microglia are highly dynamic cells with the ability to transform their morphology from ramified to amoeboid and alter their phenotypes corresponding to diverse conditions. Traditionally, macrophages and microglial cells are classified into two different phenotypes, M1 and M2. M1-microglia are proinflammatory, secreting inflammatory cytokines, chemokines, and nitric oxide (NO), which is believed to result in neuronal dysfunction and accelerate the progression of neurodegenerative diseases, such as Alzheimer’s disease (AD) and Parkinson’s disease (PD). In contrast, M2-microglia are believed to have neuroprotective functions, including increased production of interleukin (IL)-4 and neurotrophic factors, along with an increase in phagocytosis which in turn leads to clearance of cell debris and tissue damage [3, 5]. However, there is a wide spectrum of microglial activation between the two defined phenotypes [6], and microglia might even have specific neuronal functions beyond typical pro-/anti-inflammatory responses [7]. A better understanding of the interactions between microglia and other cells in the brain is therefore needed in order to design therapies to ameliorate the detrimental effects of microglial reactions in brain diseases.

The main interaction between cells occur through cellular signaling pathways including autocrine, paracrine, and endocrine processes [8] and extracellular vesicles (EVs) can be important to transport signals between cells. In fact, increasing evidence has shown EVs are considered one of the main participants in cell-to-cell communication along with having a proposed role in the spread of pathology in neurodegenerative disease [9, 10]. These vesicles are able to carry pathogen-associated and damage-associated molecular patterns that act as signals to regulate and propagate the inflammatory response [11–13]. Hence, investigation of EV trafficking under inflammatory conditions may broaden our understanding of the roles of microglia in neurodegenerative diseases, as well as their potential in therapeutic manipulation.

The secretion of EVs is a highly conserved process [14]. However, a number of studies using proteomic analysis of EVs released by various cell types, including microglia, have revealed a diverse range of markers and alteration of protein composition [15, 16]. The lack of knowledge on EV’s regulation in vitro and in vivo halts a clear understanding of EV functions in cell-to-cell communication. It is likely that different subsets of EVs have different functional properties, and trafficking of EVs is most likely modulated by specific signaling pathways.

Although consensus within the field is being reached, the classification of EVs is not easy. While different subsets of EVs are being described with increasing rate, for simplicity, we will focus on two different classes of EVs of different sizes and origins. EVs shed directly from the plasma membrane are characterized as microvesicles or ectosomes ranging from 100 to 1000 nm. Exosomes are generated within the endosomal pathway and terminate at the multivesicular endosomal body (MVB), whereby they are released upon the MVB fusing with the plasma membrane. Generally, the size of exosomes is smaller than microvesicles and below 100 nm.

A recent study has shown that the size distribution and protein composition of EVs in macrophages can be changed after bacterial infection [17]. Thus, there is a critical need for both identification of specific markers and particular signaling pathways controlling EV trafficking. The mechanism of action of EVs in microglial communication is poorly understood. In this study, we hypothesized that activation of microglia can secrete a distinct population of EV through modulation of specific signaling pathways. We investigated the dynamics of EVs from activated microglial (BV2) cells subjected to lipopolysaccharide (LPS) stimulation. We used differential ultracentrifugation to isolate EVs, including microvesicles and exosomes. EVs were then characterized in terms of size and concentration by nanoparticle tracking analysis (NTA), while the origin of EVs was indicated by western blotting using antibodies against CD63, flotillin-1, and Alix. Importantly, we also analyzed the levels of inflammatory cytokines in EVs. Secretion of EVs was altered by suppression of inflammation in microglia via inhibition of tumor necrosis factor (TNF) signaling in vivo and in vitro. Subsequently, qualitative proteomic analysis was performed to reveal a different protein composition of EVs in response to LPS challenge. Taken together, our findings provide new insights into the role of EVs in regulating microglial cell communication.

## Methods

### Cell culture

BV2, an immortalized murine microglial cell line, was cultured in growing medium containing Dulbecco’s modified Eagle medium (DMEM) (Gibco™GlutaMAX™, Thermo Fisher Scientific) supplemented with 10% heat-inactivated fetal bovine serum (FBS) and 1% penicillin/streptomycin (Thermo Fisher Scientific) in 5% CO_2_ in air at 37 °C in a humidified incubator. Cells were re-cultured every 2 days starting at a concentration of 2 × 10^5^ cells/ml in T75 flask (Sarstedt). For a large scale of EV collection, microglia were plated in T175 flask (Sarstedt). For inflammatory activation, cells were challenged with 1 μg/ml LPS (Sigma-Aldrich, Clony 0127-B8) for 12 h and then grown for 12 h in serum-free media prior to collection of EVs. For TNF inhibition experiment, microglia were plated in growing medium either with 1 μg/ml LPS, 200 ng/ml etanercept, or both for 12 h. EVs were collected from serum-free media 12 h after treatment.

### Animals

Adult male C57BL/6 mice (between 7 and 8 weeks of age, *n* = 20) were purchased from Taconic Ltd. (Ry, Denmark) and transferred to the Laboratory of Biomedicine, University of Southern Denmark, where they were allowed to acclimatize for 7 days prior to surgery. TNF knockout (TNF-KO) breeding couples were originally purchased from The Jackson Laboratory and transferred to the Laboratory of Biomedicine where they were established as a colony. Animals were housed under diurnal lighting conditions and given free access to food and water [18]. All animal experiments were performed in accordance with the relevant guidelines and regulations approved by the Danish Animal Ethical Committee (numbers 2011/561-1950 and 2013-15-2934-00924).

### Induction of experimental stroke, permanent middle cerebral artery occlusion (pMCAO)

The distal part of the left middle cerebral artery was permanently occluded under Hypnorm and Dormicum anesthesia (fentanyl citrate (0.315 mg/ml; Jansen-Cilag) and fluanisone (10 mg/ml; Jansen-Cilag, Birkerød, Denmark), and midazolam (5 mg/ml; Hoffmann- La Roche, Hvidovre, Denmark)), respectively. After surgery, mice were injected subcutaneously with 1 ml of 0.9% saline and allowed to recover in a 25 °C controlled environment. Mice surviving for 5 days were returned to the conventional animal facility after 24 h. For post-surgical analgesia, mice were treated with 0.001 mg/20 g buprenorphine hydrochloride (Temgesic, Schering-Plough, Ballerup, Denmark) three times at 8-h intervals, starting immediately prior to surgery. Mice were allowed to survive for 1 day (immunofluorescent staining and cytokine measurement) or 5 days (EV analysis) whereafter they were killed using either an overdose of pentobarbital (200 mg/ml) containing lidocaine (20 mg/ml) (Glostrup Apotek, Glostrup, Denmark) and perfused through the left ventricle using 4% paraformaldehyde (PFA) or killed by cervical dislocation. The blood and brains were collected for further analysis.

### Extracellular vesicle isolation procedure and transmission electron microscopy (TEM)

For isolation of EVs, cells were cultivated in growing medium DMEM and then deprivation of serum for a period of 12 h. The media was then collected and subjected to a series of low-speed centrifugation steps (500×*g* for 10 min, 2000×*g* for 10 min, and 10,000×*g* for 30 min) at 4 °C in order to remove cells and cellular debris. The supernatant was then collected in centrifuge tubes (Beckman Coulter) and spun at 100,000×*g* for 70 min before the resultant EV pellet was washed in a large volume of phosphate-buffered saline (PBS) before repeating the 100,000×*g* spin. The pellets containing EVs were resuspended in 20 μl of PBS and stored at 4 °C or long term at − 20 °C. For electron microscopic analysis, samples of EVs were fixed with an equal volume of 2% PFA and loaded onto Formvar/carbon-coated electron microscopic grids. EVs were observed under TEM at 80 kV. TEM was carried out at Lund University Bioimaging Center.

### Measurement of extracellular vesicles size by nanoparticle tracking analysis (NTA)

The size and total number of EVs were measured by using NanoSight LM10 (Malvern, UK) with the technology of Nanoparticle Tracking Analysis (NTA). In liquid suspension, particles undergo Brownian motion together with light scattering properties, the size distribution and concentration of EVs samples can be obtained [17]. Samples were diluted with distilled water to obtain optimal concentration for detection (10^6^–10^9^ particles/ml) and injected with a continuous syringe system for 30 s × 5 times at speed 50 μl/min. Data acquisition was undertaken at ambient temperature and measured 5 times by NTA. Data were analyzed with NTA 2.2 software (Malvern, UK) with minimum expected particle size 10 nm.

### Western blot analysis

Cell pellets and EVs were lysed in RIPA buffer (Sigma-Aldrich) supplemented with proteinase inhibitors (Thermo Scientific) and PhosphoStop (Roche Diagnostics GmbH). The concentration of cell lysates was determined using bicinchoninic acid assay (BCA) (Thermo Scientific), while concentrations obtained using NanoSight were utilized to ensure even loading of EVs. Samples were loaded onto 4–20% Mini-Protean TGX Precast Gels (Bio-Rad) and then transferred to Nitrocellulose membranes (Bio-Rad) using Trans-Blot Turbo System (Bio-Rad). Membranes were incubated with following primary antibodies: Alix (Cell Signaling; 1:1000), flotillin-1 (Cell Signaling; 1:1000), CD63 (Santa Cruz Biotechnology; 1:1000), inducible nitric oxide synthase (iNOS) (Santa Cruz Biotechnology; 1:3000), NLRP3 (Adipogen; 1:1000) and pro-caspase1 (Adipogen; 1:1000). All secondary antibodies were horse-radish protein (HRP) conjugated (Vector; 1:5000 or 1:10000). Protein bands were detected using Clarity Western ECL Substrate (Bio-Rad) or Pierce™ ECL Western Blotting Substrate (ThermoFisher), and imaged on Bio-Rad ChemiDoc XRS+. Protein levels were normalized to beta-actin (Sigma-Aldrich; 1:15,000). Image lab™ software (Bio-Rad) was used to analyze the results.

### Multiplex cytokine enzyme-linked immunosorbent assay (ELISA)

The concentrations of different cytokines in EVs and in isolated media as well as serum from mice were measured with the MSD Mouse Proinflammatory V-Plex Plus Kit (Interferonγ (IFNγ), IL-1β, IL-2, IL-4, IL-5, IL-6, IL-10, IL-12p70, TNF, C-X-C motif chemokine ligand 1 (KC/GRO), Mesoscale) using a QuickPlex SQ120 Plate Reader (Mesoscale Discovery, Rockville, USA) according to the manufacturer’s instructions. The data was analyzed with MSD Discovery Workbench software. The levels of cytokines in EVs and media were normalized to each samples total protein content in cell lysates. In total, 6 independent EV samples were analyzed; however, those samples that were under the lowest detection limit were removed from the statistical analysis.

### Immunohistochemistry

Immunofluorescent double labeling for TNF and CD11b was performed on 16-μm thick, cryostat-cut tissue sections from C57BL/6 mice with 1-day survival after pMCAO as previously described in detail [18, 19].

### Extracellular vesicle fluorescent labeling

Following isolation, EVs were labeled with PKH67 Green Fluorescent Cell Linker Midi Kit for General Cell Membrane Labeling (Sigma-Aldrich) according to the manufacturer’s instructions. Briefly, EVs were resuspended in 1 ml PBS before 1 ml of Diluent C supplemented 4 μl PKH67 dye. Samples were incubated at room temperature for 4 min prior to the addition of 2 ml of 1% bovine serum albumin (BSA) (VWR International) to bind excess dye. Samples were then supplemented with 5 ml PBS and placed in 300 kDa Vivaspin filters (Sartorius Stedim Biotech GmbH, Goettingen, Germany), prior to centrifugation for 5 min at 4000×*g* to remove excess dye. This process was repeated a further two times, followed by a further two washes in a clean filter with DMEM (Thermo Fisher Scientific) in place of PBS. The same procedure minus EVs was carried out as control.

### TNF inhibition on dynamics of extracellular vesicle trafficking

PKH67-labeled EVs (2 × 10^10^ particles/ml) were incubated with BV2 cells as indicated previously. After 12 h incubation, cells were washed three times with PBS and one time with 1 M NaCl prior to fixation with 4% PFA for 20 min on ice. Cells were then imaged by fluorescence microscope (Olympus IX71) at × 20 magnification and images processed using Cellsens Standard version 1.6 software (Olympus). Vesicle uptake was analyzed by measuring fluorescent intensity using ImageJ software (National Institutes of Health).

### Mass spectrometry

Mass spectrometry was carried out on an Orbitrap Fusion Tribrid MS system (Thermo Scientific) equipped with a Proxeon Easy-nLC 1000 (Thermo Fisher). Injected peptides were trapped on an Acclaim PepMap C18 column (3-μm particle size, 75-μm inner diameter × 20 mm length). After trapping, gradient elution of peptides was performed on an Acclaim PepMap C18 column (100 Å 3 μm, 150 mm, 75 μm). The outlet of the analytical column was coupled directly to the mass spectrometer using a Proxeon nanospray source. The mobile phases for liquid chromatography (LC) separation were 0.1% (*v*/*v*) formic acid in LC-mass spectrometry grade water (solvent A) and 0.1% (*v*/*v*) formic acid in acetonitrile (solvent B). Peptides were first loaded with a constant pressure mode with a flow rate of solvent A onto the trapping column. Subsequently, peptides were eluted via the analytical column at a constant flow of 300 nl/min. During the elution step, the percentage of solvent B increased from 5 to 22% in the first 20 min, then increased to 32% in 5 min and finally to 98% in a further 2 min and was keeping it for 8 min. The peptides were introduced into the mass spectrometer via a Stainless steel emitter 40 mm (Thermo Fisher) and a spray voltage of 1.9 kV was applied. The capillary temperature was set at 275 °C.

Data acquisition was carried out using a top N-based data-dependent method with cycle time of 3 s. The master scan was performed in the Orbitrap in the range of 350–1500 mass to charge ratio (*m/z*) at a resolution of 60,000 full width at half-maximum (FWHM). The filling time was set at maximum of 50 ms with limitation of 4 × 10^5^ ions. In a second stage of tandem mass spectrometry (MS/MS) ion trap collision-induced dissociation was acquired using parallel mode, filling time maximum 300 ms with limitation of 2 × 10^3^ ions, a precursor ion isolation width of 1.6 *m/z* and resolution of 15,000 FWHM. Normalized collision energy was set to 35%. Only multiply charged (2^+^ to 5^+^) precursor ions were selected for MS/MS. The dynamic exclusion list was set to 30 s and relative mass window of 5 ppm.

### Bioinformatic analysis

Gene Ontology (GO) classifications and enrichments were performed using FunRich [20]. The identified proteins were compared with web tool Exocarta database and also with the Top100 exosomal proteins from the database [21].

### Data analysis

MS/MS data were searched with PEAKS (7.5). UniProt *Mus musculus* (house mouse, including 16,792 sequences) was used with non-tryptic specificity allowing up to 3 missed cleavages. A 15 ppm precursor tolerance and a 0.1 Da fragment tolerance were used. Oxidation (M) and deamidation (NQ) were treated as dynamic modification and carbamidomethylation (C) as a fixed modification. Maximum post-translational modification per peptide was 2. Search results were filtered by using 1% false discovery rate and 2 unique peptides.

The rest was evaluated using either unpaired *t* test or one-way ANOVA followed by Tukey’s test for multiple comparisons. All statistical analysis was done using the GraphPad Prism 7.0 software for Macintosh (GraphPad Software, San Diego, CA, USA). Data are presented as means ± SD. A confidence interval of 95% was set as significant. The exact *P* values are given in the figure legends. Figures were organized using Adobe Illustrator.

## Results

### Proinflammatory responses from LPS-stimulated BV2 microglial cells

**Fig. 1:**
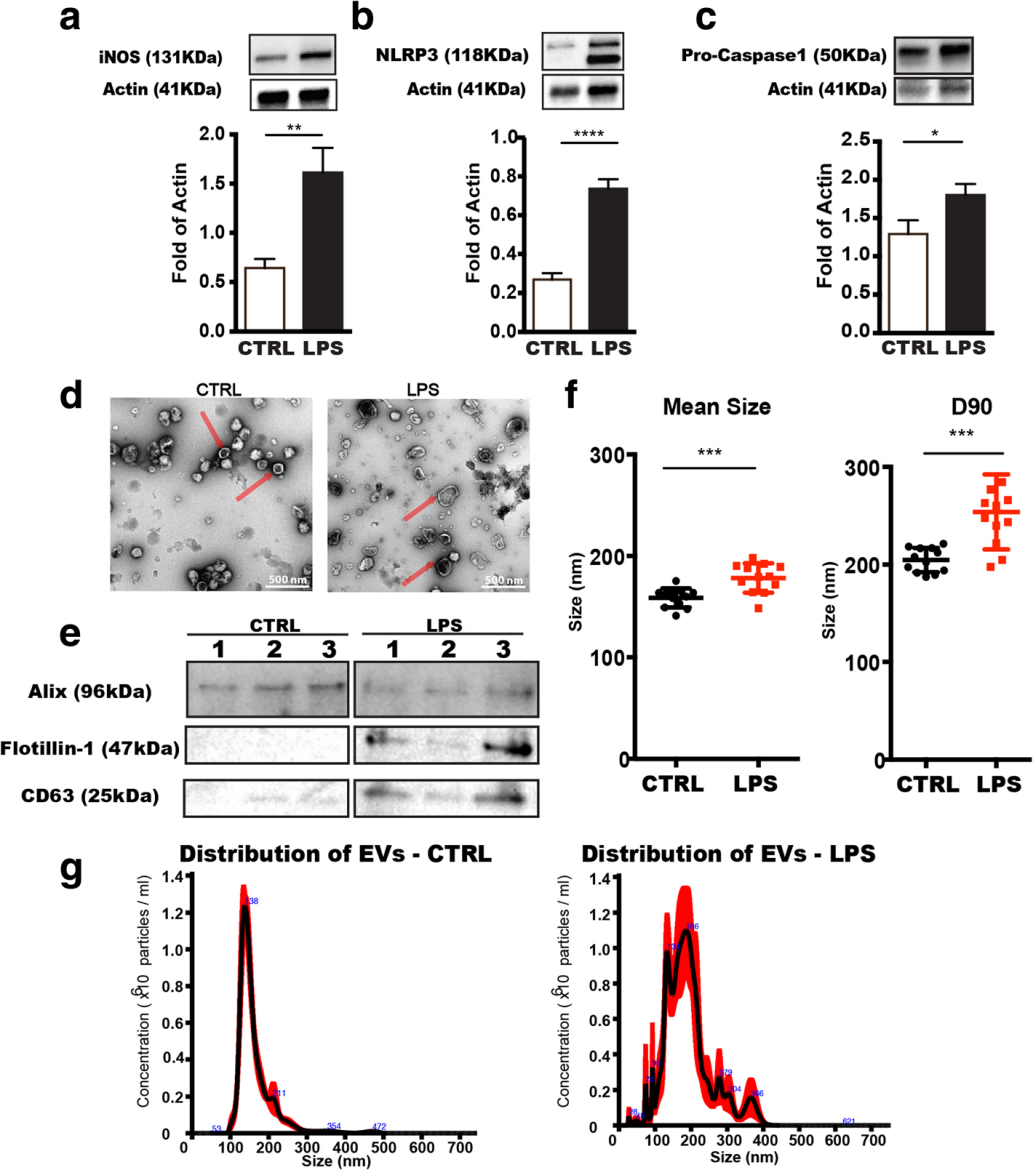
Characterization of microglia-derived extracellular vesicles. Microglia (BV2) were activated by treatment with LPS for 12 h before extracellular vesicles were isolated from the media of both LPS-treated (LPS) or control (CTRL) cells. **a** Western blot analysis of protein expression levels of iNOS in cell lysates from control and activated microglia with LPS stimulation (Mean ± SD, *n* = 5). **b**, **c** Components of the inflammasome, NLRP3 and pro-Caspase 1, were measured by western blot in cell lysates with representative pictures of blots (Mean ± SD, *n* = 5). **d** Representative TEM imaging of extracellular vesicle populations from CTRL and LPS-derived microglia. The imaging illustrates heterogeneity and sphere structure of extracellular vesicles. Typical microvesicles are pointed with red arrows. Scale bars: 500 nm. **e** Western blots showed alterations of expression levels of vesicle markers indicated different origins of extracellular vesicles from CTRL and LPS. Three biological independent samples were blotted in each condition. **f** The size of extracellular vesicles was determined in diameter from CTRL and LPS-treated microglia. The mean size shows the average diameter of extracellular vesicles in samples (*n* = 12). D90 demonstrates the upper limit of extracellular vesicles size in 90% of the population (*n* = 12). **g** Representative histograms of extracellular vesicles size distributions collected from CTRL and LPS conditions. Sample from LPS condition was diluted 25 times more than control to obtain similar concentration of EVs to demonstrate size distribution. Concentrations (× 10^6^ particles/ml) by size (nm) of recorded extracellular vesicles are showed. The major subpopulations in EV samples are indicated with digitals showing the mean diameter of extracellular vesicles. Histograms were generated from five independent measurements by NTA 2.2 software (Unpaired *t* test, *P < 0.05; ***P < 0.001; ****P < 0.0001)

First, we examined the activation of BV2 cells after 12 h culture in the presence of 1 μg/ml LPS followed by deprivation of serum for 12 h to elicit a strong inflammatory reaction. The inflammatory enzyme iNOS is expressed by activated microglia [22] and as expected, the level of iNOS was significantly increased in cells upon LPS stimulation (Fig. 1a). Moreover, the protein levels of other important inflammatory mediators, NLRP3 (Nod-like receptor protein 3) and pro-caspase1 (involved in the maturation, production, and release of IL-1β and IL-18 [23]) were found to be elevated considerably (Fig. 1b, c). According to our previous studies, the viability of BV2 cells is not affected by LPS activation [22, 24]. These results suggest that an activated pro-inflammatory status of microglia remained over the 12 h EV collection period following LPS treatment.

### Changes in EV size distribution in response to LPS activation

Transmission electron microscopy (TEM) was performed to visualize microglial-derived EVs (Fig. 1d). Images from control condition and LPS treatment revealed heterogeneous populations of EVs from microglia in the range between 100 and 1000 nm in diameter. LPS treatment seemed to induce microglial release of larger EVs, around 200–300 nm in diameter.

To further characterize EVs released under pro-inflammatory condition by LPS treatment, we compared size distribution of EVs derived from non-activated and LPS-activated microglia using Nanoparticle Tracking Analysis (NTA). The range of EVs detected with NTA was from 50 to 700 nm. Different size subpopulations of EVs were observed in EVs released from activated and non-activated microglial cells (Fig. 1f). According to the diameter of EVs, we can classify them into two subpopulations: one ranging from 50 to 100 nm can be considered as exosomes and another subpopulation with size exceeding 100 nm can be regarded as microvesicles (MVs). As can be seen in Fig. 1f, the population of MVs ranging from 300 to 400 nm has a higher frequency in the LPS-activated EVs. We found EVs released from LPS-activated cells to be significantly larger (178 ± 5.66 nm) compared to the size of EVs from control cultures (159 ± 4.95 nm) (Fig. 1f; *p* < 0.001). D90 measurement shows the upper limit of 90% measured particles, and in control condition, the D90 value for EVs was 205 ± 3.61 nm, whereas the value was 254 ± 11.06 nm in LPS-activated samples (Fig. 1f; *p* < 0.001). These results revealed that LPS-stimulated microglia release larger EV populations compared to control (Fig. 1g). EV samples were blotted for different EV markers including a marker for plasma membrane (flotillin-1) and an endosomal marker (Alix) as well as the EV marker CD63 for MVB (Fig. 1e) to elucidate the subcellular origin of the EVs [25]. We observed that in EVs released from microglia after LPS activation had a higher ratio of flotillin-1 and CD63 when compared to those from non-activated cells when loading equal amounts of EVs in each lane, suggesting altered EV biogenesis and release after an inflammatory stimulus.

### Increased production of TNF and IL-6 in EVs upon LPS activation

**Fig. 2:**
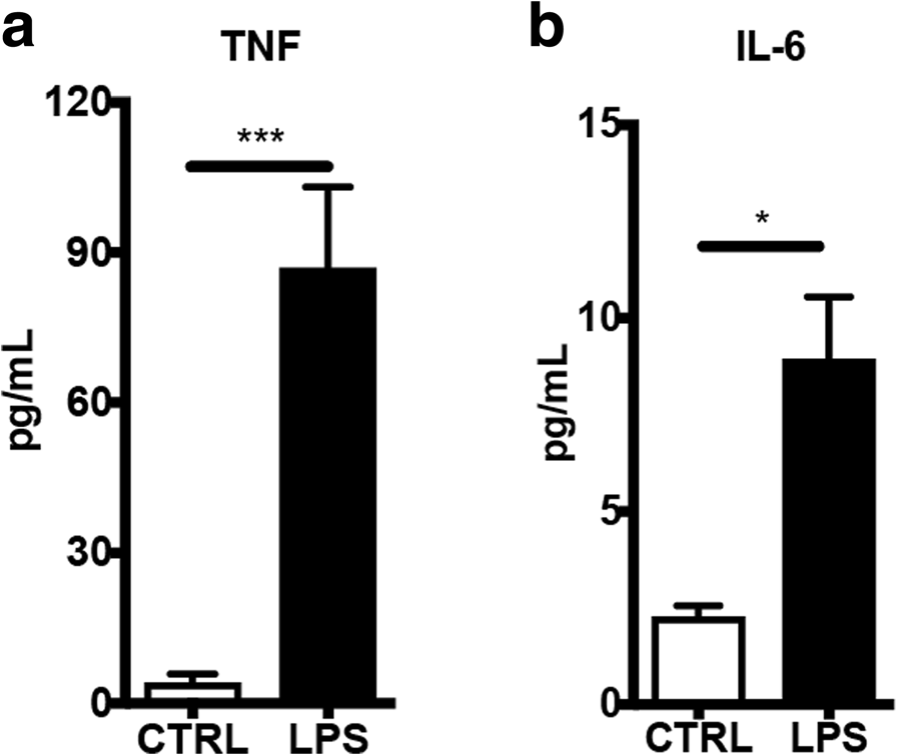
Increased levels of proinflammatroy cytokines in microglia-derived extracellular vesicles upon LPS activation. The levels of cytokines were analyzed by multiplex ELISA plate. **a** Bar graph shows a significant upregulation of TNF (*n* = 3). **b** Bar graph shows a significant upregulation of IL-6 (*n* = 6)(Mean ± SD, Unpaired *t* test, *P < 0.05; ***P < 0.001)

Next, we studied the cytokine release from LPS-activated BV2 microglia to evaluate the free concentration of released cytokines and the cytokine concentration in EVs. Culture medium from activated and non-activated microglia was collected, and EVs were isolated from equal amounts of medium. EVs, along with the EV-depleted media, were then subjected to analysis by multiplex ELISA. Out of ten inflammatory cytokines analyzed, the levels of two pro-inflammatory cytokines, TNF and IL-6, were found to be significantly increased in EVs from activated microglia (Fig. 2a, b). TNF and IL-6 are two representative pro-inflammatory cytokines produced by microglia related to neurodegenerative diseases [3]. Notably, there was also a significant upregulation in the concentration of these two cytokines in medium (Additional file 1). However, other pro-inflammatory cytokines such as IL-5 and IL-1β were found to be increased only in the medium, but not in EVs (Additional files 1 and 2). Importantly, the level of TNF was much higher increased, 22-fold, compared to 5-fold in IL-6. Thus, we further investigated the effect of TNF in regulation of EV release in the following study.

### Reduction of EVs by inhibition of inflammation via TNF pathway

**Fig. 3:**
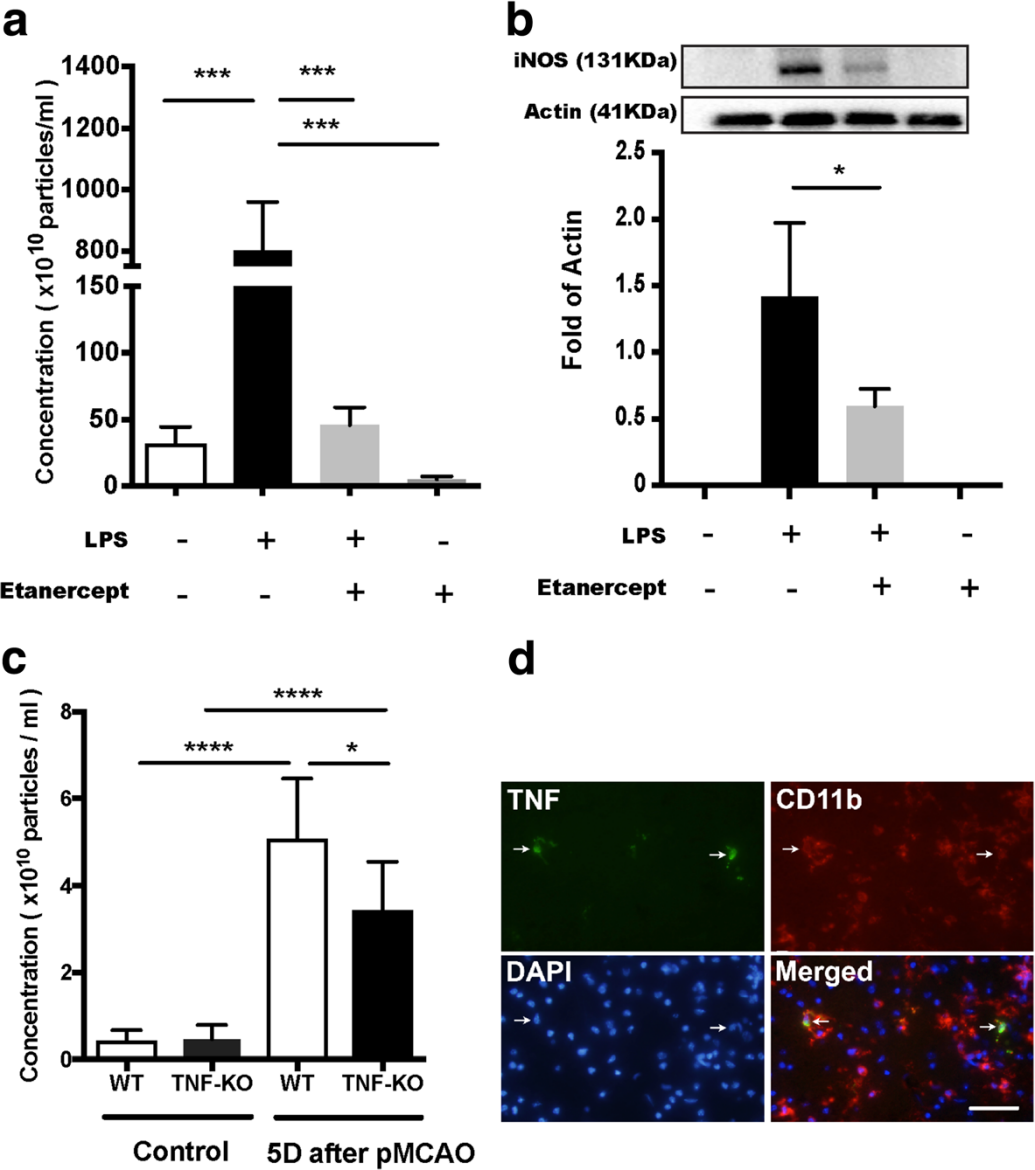
Reduction in the number of microglia-derived extracellular vesicles by inhibition of TNF signaling pathway. Extracellular vesicles were visualized by TEM. Concentrations of extracellular vesicles in conditional media and plasma from mice were measure by NTA. **a** Comparison of extracellular vesicles concentrations in different conditional media. BV2 microglia cell line was treated with either LPS (1 μg/ml) or etanercept (200 ng/ml) or in the presence of both. Control (CTRL) was cells without any treatment (Mean ± SD, one-way ANOVA, ***P < 0.001, *n* = 3). **b** Expression levels of iNOS in BV2 cells previously treated with different conditions were analyzed by western blot. iNOS bands were not able to be visualized in the conditions of control and etanercept due to out of the detection limit (Mean ± SD, Unpaired t-test, *P < 0.05, *n* = 4). **c** Bar graph shows a comparison of the amount of extracellular vesicles in plasma from mice (WT, n = 6; TNF-KO, n = 6) without surgery and mice (WT, *n* = 9; TNF-KO, *n* = 8) 5 days after a stroke model, permanent middle cerebral artery occlusion (pMCAO) (Mean ± SD, one-way ANOVA followed by Tukey’s test for multiple comparisons, *P < 0.05, ***P < 0.001). **d** The brain section in area of infarct from C57BL/6 mouse subjected to pMCAO stained with anti-TNF (green) and anti-CD11b (red), nuclei stained with DAPI. TNF+ cells are also stained for CD11b indicated with arrow; scale bar: 20 μm

In view of the specific increase of TNF in the EVs after LPS-stimulation, we set out to further characterize the role of TNF in the microglial release of EV. To investigate the mechanistic basis for EV regulation on LPS signaling in microglial cells, we quantified the number of EVs released from microglia after TNF inhibition with etanercept upon LPS activation. Notably, activated microglia secreted a 30-fold increase in the number of EVs under LPS stimulation (Fig. 3a). In contrast, the effect of LPS on EV release was completely attenuated down to control levels using etanercept (200 ng/ml), a TNF inhibitor that blocks both soluble and transmembrane forms of TNF (Fig. 3a). In the presence of the TNF inhibitor, the amount of EVs released from cells was also reduced compared with control condition (Fig. 3a). Next, we wanted to understand whether the reduction of EV is caused by decreased EV uptake through inhibition of TNF. Thus, we assessed the capability of EV uptake under different conditions as indicated above. We found no significant difference between the conditions on internalization of PHK76-labeled EVs, indicating the reduction of EVs was due to TNF treatment on EV release not by affecting EV uptake/turnover (Additional file 3).

Next, we evaluated the degree of inflammatory status in microglia in relation to the amount of EVs secreted. To that aim, the level of iNOS was analyzed by western blot on cells previously treated in different conditions. We found two-fold reduction in iNOS levels following TNF inhibition. Interestingly, the reduction of EVs released upon TNF inhibition was reduced 16-fold, suggesting that TNF signaling is particularly important when it comes to reducing the number of EV released in proinflammatory activation of microglia (Fig. 3a). Together, these results implicate that the secretion of EVs is dramatically impeded by TNF inhibition in LPS-activated microglia, which is only partly associated to an overall reduction in the inflammatory status.

### Evaluation of systemic inflammation in mice after focal cerebral ischemia

**Fig. 4:**
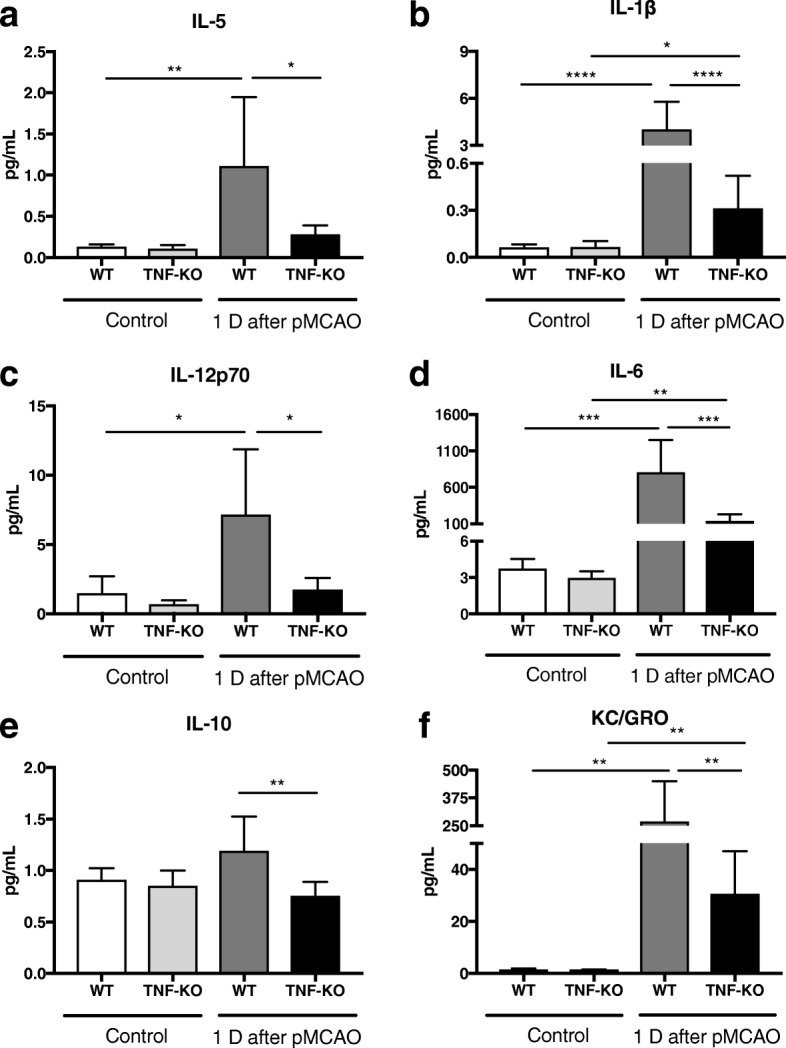
Systemic inflammation in mice after focal cerebral ischemia. The levels of cytokines in serum were analyzed by multiplex ELISA from WT and TNF-KO mice without manipulation and 1 day after focal cerebral ischemia. **a** and **c** Bar graphs show significant upregulation of IL-5 and IL-12p70 in WT mice after pMCAO. **b**, **d**, **f** Bar graphs show significant upregulation of IL-1β, IL-6, and KC/GRO in both types of mice subjected to pMCAO. **e** Bar graph shows remarkable reduction of IL-10 in TNF-KO mice compared with WT mice 1 day after pMCAO. (Mean ± SD, one-way ANOVA followed by Tukey’s test for multiple comparisons, *n* = 3–6, *P < 0.05; **P < 0.01; ***P < 0.001; ****P < 0.0001)

As the level of TNF has shown to be upregulated in EVs under LPS activation in vitro, we next studied whether the secretion of EVs in vivo was affected by complete ablation of TNF signaling in a strong neuroinflammatory situation. To this purpose, we chose an experimental stroke model, permanent middle cerebral artery occlusion (pMCAO), as an in vivo inflammatory model. Experimental stroke in rat and mouse is known to induce neuroinflammation in the brain [26, 27] as well as alter the inflammatory response in the periphery [28, 29]. Our earlier study has revealed significant increase of TNF receptors, toll-like receptor (TLR) 2 and IL-1β at mRNA levels in wild type (WT) and TNF-KO mice 1 day after pMCAO, indicating inflammation occurred in the brain [18]. We also wanted to assess systemic inflammatory response in mice after pMCAO. Therefore, we measured levels of different cytokines in serum using Multiplex ELISA from WT and TNF-KO mice without manipulation and 1 day after pMCAO. TNF expression levels were statistically elevated in WT at day 1 and unchanged in TNF-KO mice (Additional file 4). IL-5 and IL-12p70 were also found statistically elevated in WT mice 1 day after manipulation, but not in TNF-KO (Fig. 4a, c). While in the case of IL-1β, IL-6, and KC/GRO, expression levels were considerably increased 1 day after pMCAO in both types of mice compared with unmanipulated mice (Fig. 4b, d, f). Notably, such increases induced by pMCAO were significantly attenuated by deficiency of TNF in mice. Levels of IL-10 were remarkably lower in TNF-KO mice compared with WT mice after pMCAO, but not before (Fig. 4e). Levels of IL2, IL-4, and IFNγ were remained at baseline levels at day 1 in both types of mice subjected to pMCAO (Additional file 4). In conclusion, we found clear evidence of significant upregulation of proinflammatory cytokines at day 1 after pMCAO in both types of mice. However, in TNF-KO mice, such inflammation induced by pMCAO was remarkably attenuated at day 1 after stroke.

### Decreased EVs in TNF knockout mice after focal cerebral ischemia

Given the impact of pMCAO on systemic inflammation in WT and TNF-KO mice, we examined microglia activation in the brain. We observed that TNF co-localized with the microglial marker, CD11b, in the peri-infarct area 1 day after pMCAO (Fig. 3d), which shows that focal cerebral ischemia could induce an initial phase of microglia activation involving TNF signaling and tissue injury, as we have shown before [30]. Moreover, the volume of infarct was also assessed in WT mice and TNF-KO mice 5 days after pMCAO in the previous study, which has shown that the injury was significantly larger in TNF-KO mice than WT mice [18]. Previously, we have shown that the infarct volume correlates with the number of EVs in plasma after this stroke model [31]. Thus, we analyzed the number of EVs in the plasma of TNF-KO mice after pMCAO. The production of EVs increased from both genotypes 5 days after pMCAO indicating inflammation occurred, which in line with our previous findings in vitro (Fig. 3a, c). Importantly, we found here that a complete ablation of TNF successfully reduced the counts of EVs in the plasma of TNF-KO mice 5 days after induction of permanent focal cerebral ischemia (Fig. 3c), but not at day 1 (Additional file 5). These results are consistent with our in vitro data and indicate the number of EVs is related to an inflammatory event and that TNF signaling is important in the mechanism regulating EV release.

### Identification of microglial EVs proteins

**Fig. 5:**
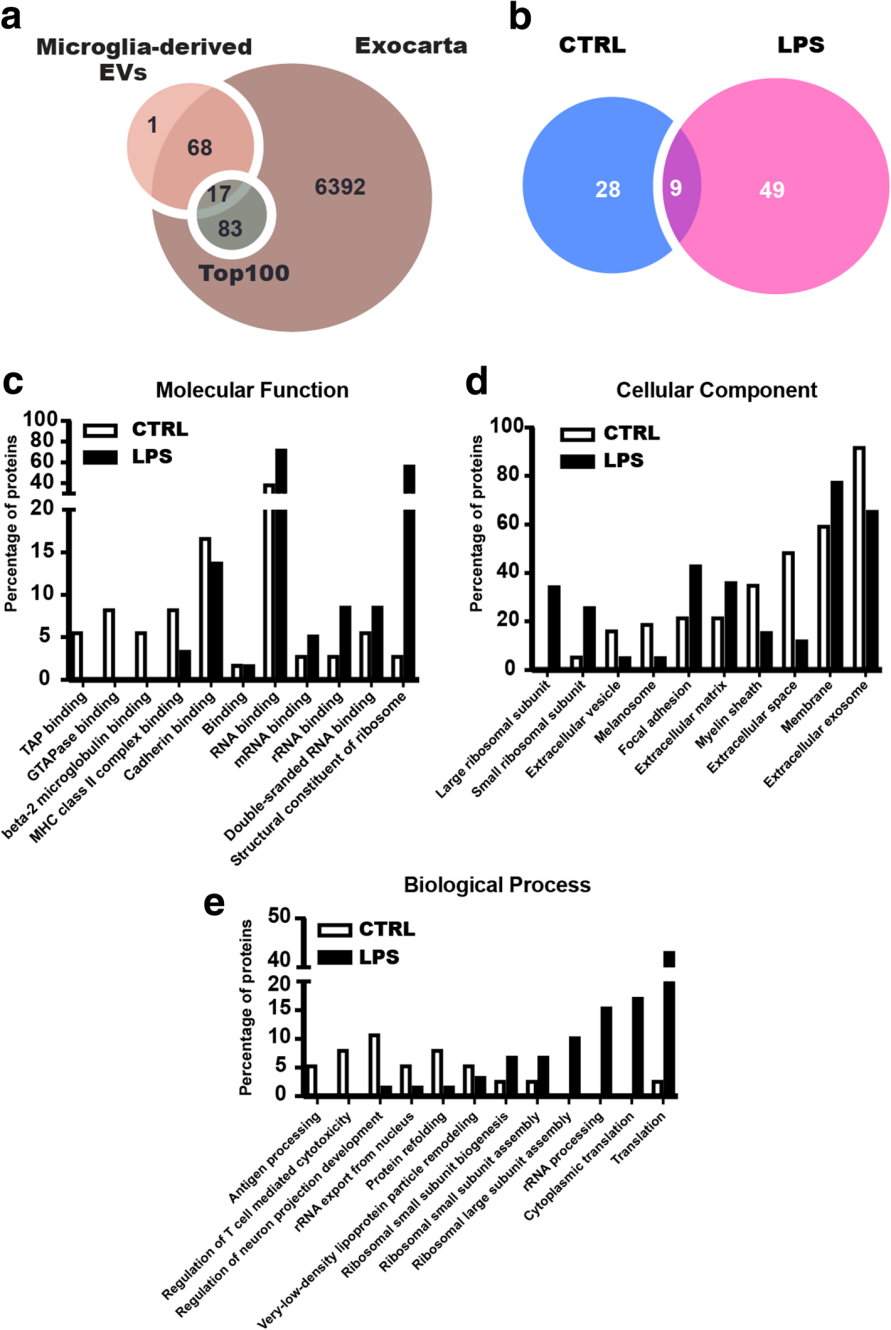
Bioinformatic analysis of the identified proteins in BV2 microglia-derived extracellular vesicles. Gene ontology (GO) analysis of the identified proteins was performed using Exocarta based software FunRich. The comparison was carried on proteins expressed exclusively in each condition. **a** Comparison of 86 identified proteins from microglial extracellular vesicles with online database Exocarta and the top 100 proteins commonly reported in the same database. **b** Comparison of the number of proteins identified and quantified in control microglia and LPS-activated microglia derived extracellular vesicles samples. **c** Bioinformatic analysis from FunRich shows comparison of GO analysis on Molecular Function on proteins from CTRL and LPS-activated microglia released extracellular vesicles (GO terms are with *P* < 0.01). **d** Bioinformatic analysis from FunRich shows comparison of GO analysis on Cellular Component on proteins from CTRL and LPS-activated microglia released extracellular vesicles (GO terms are with *P* < 0.01). **e** Bioinformatic analysis from FunRich shows comparison of GO analysis on Biological Process on proteins from CTRL and LPS-activated microglia released extracellular vesicles (GO terms are with *P* < 0.01)

To further elucidate cellular communication by EVs under inflammation, we set out to identify proteins in association to EVs released by BV2 cells. To this aim, proteomic analysis was performed using mass spectrometry on EV samples from LPS-activated and control microglia. Biological independent duplicates of EVs were pooled together and analyzed. In total, 86 proteins were identified with two peptides confirmed and high confidence, as -10lgP is set for 20 as threshold, using PEAKS analysis program (Tables 1 and 2) [32]. In total, 37 proteins from control and 58 proteins from LPS-stimulated microglia were successfully mapped with UniProt database. Among them, we found 9 proteins in common (Table 3) and 49 specific proteins present in LPS condition. The analysis showed enzymes, chaperones, ribosomal structure proteins, and membrane receptors previously reported in other immune cells in our samples (Fig. 5 d, e). Of these, the majority of the identified proteins were associated with RNA binding, and more than half of them had Gene Ontology annotations related to membrane and extracellular exosome (Fig. 5c, d). The identified proteins were also compared with the ExoCarta database, which has exosomal proteins identified from previous publications [21]. In our samples, only one protein has not been reported in ExoCarta database (Fig. 5a). Compared with top 100-ranked proteins presented in the database, we identified 17 of them in our study (Fig. 5a). Notably, 89.5% proteins from this study have not been reported before and first identified in microglia (Table 4), suggesting that these cells are releasing specific EVs.

**Table 1:** List of proteins identified in control EV samples

Protein ID	Gene name	Protein name	Score
P08226	APOE	Apolipoprotein E	506
P21956	MFGE8	Lactadherin	136
P21460	CST3	Cystatin-C	123
Q9WU78	PDCD6IP	Programmed cell death 6-interacting protein	121
P11152	LPL	Lipoprotein lipase	107
Q8VDN2	ATP1A1	Sodium/potassium-transporting ATPase subunit alpha-1	105
P62737	ACTA2	Actin, aortic smooth muscle	84
Q61753	PHGDH	D-3-phosphoglycerate dehydrogenase	80
P07901	HSP90AA1	Heat shock protein HSP 90-alpha	80
P10605	CTSB	Cathepsin B	75
P01942	HBA	Hemoglobin subunit alpha	73
P63017	HSPA8	Heat shock protein 8	71
Q62419	SH3GL1	Endophilin-A2	70
Q8R366	IGSF8	Immunoglobulin superfamily member 8	69
Q68FD5	CLTC	Clathrin heavy chain 1	67
P10923	SPP1	Osteopontin	64
Q61207	PSAP	Prosaposin	64
P06869	PLAU	Urokinase-type plasminogen activator	62
Q8CGP5	HIST1H2AF	Histone H2A type 1-F	61
P52480	PKM	Pyruvate kinase	59
P17182	ENO1	Alpha-enolase	56
P04104	KRT1	Keratin, type II cytoskeletal 1	50
P68369	TUBA1A	Tubulin alpha-1A chain	47
P01887	B2M	Beta-2-microglobulin	45
P09405	NCL	Nucleolin	44
P09055	ITGB1	Integrin beta-1	42
P01901	H2-K1	H-2 class I histocompatibility antigen, K-B alpha chain	41
P01899	H2-D1	H-2 class I histocompatibility antigen, D-B alpha chain	36
P29341	PABPC1	Polyadenylate-binding protein 1	41
P06797	CTSL	Cathepsin L1	38
P10852	SLC3A2	4F2 cell-surface antigen heavy chain	37
P08905	LYZ2	Lysozyme C-2	34
P10126	EEF1A1	Elongation factor 1-alpha 1	28
Q61937	NPM1	Nucleophosmin	27
Q9DBJ1	PGAM1	Phosphoglycerate mutase 1	25
P28798	GRN	Granulins	24
P14206	RPSA	40S ribosomal protein SA	24

Proteins retrieved in control extracellular vesicle samples from mass spectrometry. Protein ID and gene name are according to Uniprot Knowledgebase. Score values were obtained using MASCOT. The Score shows how well the observed protein matches to the stated protein in the database. Only protein identifications supported by at least two high confident peptides (confidence > 95%) were considered

**Table 2:** List of proteins identified in LPS EV samples

Protein ID	Gene name	Protein name	Score
P68372	TUBB4B	Tubulin beta-4B chain	258
P62242	RPS8	40S ribosomal protein S8	201
P47963	RPL13	60S ribosomal protein L13	181
Q9CZX8	RPS19	40S ribosomal protein S19	178
P15864	HIST1H1C	Histone H1.2	172
Q8VEK3	HNRNPU	Heterogeneous nuclear ribonucleoprotein U	148
Q9CR57	RPL14	60S ribosomal protein L14	146
P63276	RPS17	40S ribosomal protein S17	138
P11152	LPL	Lipoprotein lipase	130
P16858	GAPDH	Glyceraldehyde-3-phosphate dehydrogenase	128
P68369	TUBA1A	Tubulin alpha-1A chain	123
P62082	RPS7	40S ribosomal protein S7	106
Q9D8E6	RPL4	60S ribosomal protein L4	101
P35979	RPL12	60S ribosomal protein L12	101
P14131	RPS16	40S ribosomal protein S16	100
P70696	HIST1H2BA	Histone H2B type 1-A	100
P12970	RPL7A	60S ribosomal protein L7a	98
P60710	ACTB	Actin, cytoplasmic 1	94
Q9CXW4	RPL11	60S ribosomal protein L11	85
Q9CZM2	RPL15	60S ribosomal protein L15	84
P02301	H3F3C	Histone H3.3C	83
P47911	RPL6	60S ribosomal protein L6	83
P43276	HIST1H1B	Histone H1.5	83
Q8CGP5	HIST1H2AF	Histone H2A type 1-F	82
P14115	RPL27A	60S ribosomal protein L27a	74
P27659	RPL3	60S ribosomal protein L3	72
O55142	RPL35A	60S ribosomal protein L35a	70
P25206	MCM3	DNA replication licensing factor MCM3	68
P62702	RPS4X	40S ribosomal protein S4, X isoform	63
P10126	EEF1A1	Elongation factor 1-alpha 1	63
Q8BP67	RPL24	60S ribosomal protein L24	62
P14206	RPSA	40S ribosomal protein SA	62
P11499	HSP90AB1	Heat shock protein HSP 90-beta	60
P08226	APOE	Apolipoprotein E	60
P35980	RPL18	60S ribosomal protein L18	59
P63017	HSPA8	Heat shock protein 8	59
P80318	CCT3	T-complex protein 1 subunit gamma	58
P62918	RPL8	60S ribosomal protein L8	57
O08585	CLTA	Clathrin light chain A	55
Q9Z1Q9	VARS	Valine-tRNA ligase	54
P01942	HBA	Hemoglobin subunit alpha	51
P97351	RPS3A	40S ribosomal protein S3a	50
P62806	HIST1H4A	Histone H4	48
P62270	RPS18	40S ribosomal protein S18	44
P62911	RPL32	60S ribosomal protein L32	43
P14869	RPLP0	60S acidic ribosomal protein P0	41
Q9JIK5	DDX21	Nucleolar RNA helicase 2	41
P62281	RPS11	40S ribosomal protein S11	41
Q68FD5	CLTC	Clathrin heavy chain 1	40
P62717	RPL18A	60S ribosomal protein L18a	39
P86048	RPL10L	60S ribosomal protein L10-like	39
P62267	RPS23	40S ribosomal protein S23	37
P25444	RPS2	40S ribosomal protein S2	36
P14148	RPL7	60S ribosomal protein L7	31
P53026	RPL10A	60S ribosomal protein L10a	28
P62264	RPS14	40S ribosomal protein S14	28
P62908	RPS3	40S ribosomal protein S3	23
P04918	SAA3	Serum amyloid A-3 protein	23

Proteins retrieved in LPS extracellular vesicles samples from mass spectrometry. Protein ID and gene name are according to Uniprot Knowledgebase. Score values were obtained using MASCOT. The score shows how well the observed protein matches to the stated protein in the database. Only protein identifications supported by at least two high confident peptides (confidence > 95%) were considered

**Table 3:** The common proteins shared in CTRL and LPS EV samples

Protein ID	Gene name	Protein name
P08226	APOE	Apolipoprotein E
P11152	LPL	Lipoprotein lipase
P01942	HBA	Hemoglobin subunit alpha
P63017	HSPA8	Heat shock protein 8
Q68FD5	CLTC	Clathrin heavy chain 1
Q8CGP5	HIST1H2AF	Histone H2A type 1-F
P68369	TUBA1A	Tubulin alpha-1A chain
P10126	EEF1A1	Elongation factor 1-alpha 1
P14206	RPSA	40S ribosomal protein SA

Proteins retrieved in extracellular vesicle samples from mass spectrometry. Protein ID and gene name are according to Uniprot Knowledgebase

**Table 4:** New proteins found in present microglia-derived EV samples

Protein ID	Gene name	Protein name
P08226	APOE	Apolipoprotein E
P21460	CST3	Cystatin-C
Q8VDN2	ATP1A1	Sodium/potassium-transporting ATPase subunit alpha-1
P62737	ACTA2	Actin, aortic smooth muscle
Q61753	PHGDH	D-3-phosphoglycerate dehydrogenase
P10605	CTSB	Cathepsin B
P01942	HBA	Hemoglobin subunit alpha
Q62419	SH3GL1	Endophilin-A2
Q8R366	IGSF8	Immunoglobulin superfamily member 8
Q68FD5	CLTC	Clathrin heavy chain 1
P10923	SPP1	Osteopontin
Q61207	PSAP	Prosaposin
P06869	PLAU	Urokinase-type plasminogen activator
Q8CGP5	HIST1H2AF	Histone H2A type 1-F
P04104	KRT1	Keratin, type II cytoskeletal 1
P68369	TUBA1A	Tubulin alpha-1A chain
P01887	B2M	Beta-2-microglobulin
P09405	NCL	Nucleolin
P09055	ITGB1	Integrin beta-1
P01901	H2-K1	H-2 class I histocompatibility antigen, K-B alpha chain
P01899	H2-D1	H-2 class I histocompatibility antigen, D-B alpha chain
P29341	PABPC1	Polyadenylate-binding protein 1
P06797	CTSL	Cathepsin L1
P10852	SLC3A2	4F2 cell-surface antigen heavy chain
P08905	LYZ2	Lysozyme C-2
P10126	EEF1A1	Elongation factor 1-alpha 1
Q61937	NPM1	Nucleophosmin
P28798	GRN	Granulins
P14206	RPSA	40S ribosomal protein SA
P68372	TUBB4B	Tubulin beta-4B chain
P62242	RPS8	40S ribosomal protein S8
P47963	RPL13	60S ribosomal protein L13
Q9CZX8	RPS19	40S ribosomal protein S19
P15864	HIST1H1C	Histone H1.2
Q8VEK3	HNRNPU	Heterogeneous nuclear ribonucleoprotein U
Q9CR57	RPL14	60S ribosomal protein L14
P63276	RPS17	40S ribosomal protein S17
P62082	RPS7	40S ribosomal protein S7
Q9D8E6	RPL4	60S ribosomal protein L4
P35979	RPL12	60S ribosomal protein L12
P14131	RPS16	40S ribosomal protein S16
P70696	HIST1H2BA	Histone H2B type 1-A
P12970	RPL7A	60S ribosomal protein L7a
P60710	ACTB	Actin, cytoplasmic 1
Q9CXW4	RPL11	60S ribosomal protein L11
Q9CZM2	RPL15	60S ribosomal protein L15
P02301	H3F3C	Histone H3.3C
P47911	RPL6	60S ribosomal protein L6
P43276	HIST1H1B	Histone H1.5
Q8CGP5	HIST1H2AF	Histone H2A type 1-F
P14115	RPL27A	60S ribosomal protein L27a
P27659	RPL3	60S ribosomal protein L3
O55142	RPL35A	60S ribosomal protein L35a
P25206	MCM3	DNA replication licensing factor MCM3
P62702	RPS4X	40S ribosomal protein S4, X isoform
P10126	EEF1A1	Elongation factor 1-alpha 1
Q8BP67	RPL24	60S ribosomal protein L24
P14206	RPSA	40S ribosomal protein SA
P11499	HSP90AB1	Heat shock protein HSP 90-beta
P08226	APOE	Apolipoprotein E
P35980	RPL18	60S ribosomal protein L18
P63017	HSPA8	Heat shock protein 8
P80318	CCT3	T-complex protein 1 subunit gamma
P62918	RPL8	60S ribosomal protein L8
P62911	RPL32	60S ribosomal protein L32
P14869	RPLP0	60S acidic ribosomal protein P0
Q9JIK5	DDX21	Nucleolar RNA helicase 2
P62281	RPS11	40S ribosomal protein S11
P62717	RPL18A	60S ribosomal protein L18a
P86048	RPL10L	60S ribosomal protein L10-like
P62267	RPS23	40S ribosomal protein S23
P25444	RPS2	40S ribosomal protein S2
P14148	RPL7	60S ribosomal protein L7
P53026	RPL10A	60S ribosomal protein L10a
P62264	RPS14	40S ribosomal protein S14
P62908	RPS3	40S ribosomal protein S3
P04918	SAA3	Serum amyloid A-3 protein

Proteins identified in present study were compared with proteins uploaded in Exocarta database from microglial origin. Protein ID and gene name are according to Uniprot Knowledgebase

### Functional profiles of the quantified microglia EVs proteins

Next, we wanted to know the functions of the quantified proteins differentially secreted in EVs under LPS activation. The 86 quantified proteins in the microglial-derived EVs were analyzed using FunRich software [21] to validate our data referred to the Vesiclepedia Exosome Database [33]. The analysis was based on their cellular compartments to which they belong, their molecular function and the biological process in which they are involved. In the analysis, some of the proteins were annotated in more than one cellular component, molecular function, and biological process.

Firstly, enrichment in pathways from membrane and extracellular exosome was observed for GO analysis on cellular component (Fig. 5d). There were large increases in proteins from LPS-stimulated EVs from ribosome, focal adhesion, extracellular matrix, and membrane (Fig. 5d). In contrast, a reduction of proteins associated with extracellular exosome was found in LPS-stimulated EVs, which is also confirmed by western blot with increased ratio of flotillin-1 and CD63 indicating more MVs released. In the categories of molecular function, the majority of the proteins were annotated to RNA binding, 38.9% in control and 72.4% in LPS (Fig. 5c). Importantly, the proteins contributed to ribosome function raised from 2.8 to 56.9% after LPS challenge (Fig. 5c). The profile of proteins in molecular function was dramatically altered after inflammatory stimulation. It is likely that microglia released a distinct population of EVs related to transduction and translation after activation of LPS. Intriguingly, the profile of EVs was peculiarly different after activation according to classification of biological process (Fig. 5e). EVs were found involved in regulation of neuron projection development at “rest” status (control), however, with activation the proteins identified in EVs shifted to ribosomal assembly and translation. The role of microglia was changed from “rest” state to “activated” state by not only production of cytokine and chemokine, but also with altered secretion of EVs, which could be directly linked to detrimental inflammatory responses related to neurodegenerative diseases.

In larger proportion, 49 proteins were exclusively present in EVs samples from LPS activation, including some cytoskeleton proteins and ribosomal proteins. Importantly, three of them were associated with inflammatory and neuropathological pathways: tubulin beta 4B (TUBB4B), heterogeneous nuclear ribonucleoprotein U (HNRNPU) and serum amyloid A 3 (SAA3). Nine proteins were shared in samples from control and LPS conditions (Fig. 5b). Proteins related to immune system process and stimuli response were detected in both samples: lipoprotein lipase (LPL), apolipoprotein E (APOE), and heat shock protein 8 (HSPA8). Notably, APOE was particular with high score in the EVs before and after LPS activation. Variants of APOE (APOE4) is known as the strongest risk factor for late onset Alzheimer’s disease [34] and has also been suggested to have a proinflammatory effect on microglia [35].

## Discussion

Compelling evidence has suggested that the activation of innate immunity and neuroinflammation play crucial roles in neurodegenerative diseases [36–38]. Microglia are an essential component in innate immunity in the CNS. Activation of microglia is followed by the initiation of intracellular machinery that leads to production of cytotoxic and proinflammatory cytokines and chemokines, which promote progression of inflammation and affect neighboring cells through different mechanisms. The secretion of proinflammatory signals can be conducted in classic secretion manner or non-canonical manner via vesicles. Previous studies have shown the important role of EVs in regulation of cytokine production on recipient cells and propagation of pathogenic proteins [11, 39].

The functions of microglia in the brain are diverse depending on stimulus and different brain regions. In response to different inflammatory/homeostatic conditions, they can be either beneficial or detrimental. Increasing evidence has suggested that there is a spectrum of activation in microglia based on the profile of secreted molecules [3]. However, the importance of EV released by microglia has not been well characterized. Thus, in the present study, we directly evaluated effects of LPS-stimulation on EVs derived from BV2 microglia, in terms of physical and biological properties. Our results demonstrate that (i) upon LPS-activation, the size distribution of EVs released from microglia increases in size, indicating larger vesicles are released under inflammatory conditions; (ii) IL-6 and in particular TNF are increasingly secreted in microglia-derived EVs after LPS challenge; (iii) inhibiting the TNF signaling pathway resulted in a robust reduction in the number of vesicles released from LPS-treated microglia and in mice subjected to pMCAO; and (iv) in response to LPS, BV2 microglia release EVs with a distinct proteomic profile related to transcription and translation.

EVs can either enhance or suppress inflammation and act as main factors to regulate inflammation and immunity [40, 41]. There is evidence showing microglia can release IL-1β, upon exposure to ATP derived from astrocytes, by shedding MVs which contain the entire machinery important for processing of it including the P2X7 receptors [42]. This is consistent with our findings that EVs contain more molecules related to transcription and translation in activated state and include a particular population of MVs budding from the plasma membrane. The mechanism underlying release of EV is still not clear. TNF can induce neurotoxicity by modulating glutamate production that results in excitotoxic neuronal death [43]. One study conducted by Wang et al. has shown that TNF promotes the release of EVs from astrocytes through increased expression of glutaminase, which convert glutamine to glutamate [44]. TNF can also induce extensively production of glutamate from microglia in an autocrine manner to cause excitotoxicity and contribute to neuronal damage [45]. Thereby, these findings together with our results suggest that microglial EV release could be potentially modulated by TNF through specifically regulated mechanisms.

Our data suggests that inhibition of TNF signaling in microglia may impact on inflammation, with an observed reduction both in the release of different cytokines and in the total number of EVs released, implying that the release of EVs in activated microglia seems to be regulated. This idea is further supported by data from our experimental stroke model that showed altered EV counts in plasma from TNF-KO mice compared to WT. These results contradict previous work that showed an increase in the amount of MVs in mice treated with TNF inhibitor for 5 days after pMCAO compared to saline-treated mice [31]. It is therefore reasonable to assume that the difference we observed is due to the different experimental set-up. A systemic administration of TNF inhibitor in the mice 30 min after surgery could merely have a transient effect on the TNF signaling pathway, whereas using the TNF-KO model is expected to be more stable to investigate and characterize long-term effects of EVs release.

From our previous study, pMCAO could initiate microglia activation and inflammation in the brain [18, 28]. Therefore, we also evaluated systemic inflammation in mice subjected to pMCAO. Although several inflammatory factors, such as IL-1β and IL-6, were significantly increased in both types of mice at day 1 after manipulation, such induction was attenuated in TNF-KO mice. Another study has shown that EVs derived from macrophages stimulated with bacterial infection are able to increase secretion of proinflammatory cytokines in recipient cells, including TNF [17]. Taken together, we can speculate that inflammatory propagation can be mitigated by a reduction in the number of microglia-derived EVs, thus halting inflammation via TNF signaling. However, this is complicated by the fact that there are two forms of TNF, soluble TNF (solTNF), which is related to neurotoxicity and inflammation, while the transmembrane TNF (tmTNF) is involved in functional recovery and neuroprotection [18, 46]. Hence, both the cellular contribution to TNF signaling and specifically the form of TNF carried in microglial EVs is important to evaluate final outcome of experiment, neuroprotective or neurotoxic. In our study, we measured total TNF in EVs including tmTNF and solTNF. However, the specific form of TNF is likely to be important for the inflammatory and cytoprotective outcome. We believe such studies investigating the specific form of TNF carried in EVs are important in future studies.

We also studied the protein composition to further characterize the EVs released under control or inflammatory conditions. Using western blot analysis, we looked for changes in the levels of either plasma membrane or endosomal markers to elucidate where these vesicles had originated. Indeed, we observed an increase in signal from plasma membrane markers when compared to endosomal markers upon LPS activation. When combined with the increase in overall size observed with NanoSight, this supports our theory that more MVs are released under inflammatory conditions.

Furthermore, our proteomic analysis indicated that the two populations of EVs were dramatically different in categories of molecular function and biological process under GO analysis. From the qualitative proteomic analysis, 86 proteins were identified with high accuracy. Compared to Exocarta database, most of the proteins have been reported in previous studies in exosomes from other cell types, where 89.5% of the quantified proteins were firstly identified in microglial EVs. The small overlap with previous studies is most likely due to a lack of studies utilizing microglia-derived EVs, with a sole study performed on EVs from the N9 microglial cell line responsible for all microglial proteins present in ExoCarta [15]. It was also clear from the FunRich analysis [20] that the proteins detected in EVs from LPS-treated cells had different functions to those from control cells, with proteins involved in RNA binding and structural components of the ribosome more prevalent in LPS-derived EVs. While the analysis on the cellular origin of the EV proteins revealed that the extracellular exosome and membrane were dominant from both conditions, it is of interest to note that EVs isolated from inflammatory condition were detected with more membrane and less exosome proteins, consistent with a shift towards MV release rather than exosome.

For the first time, our data indicates that microglia change its EVs releasing machinery after LPS activation with an increase in the overall number of EVs, and more EVs budding from the membrane. It is tempting to speculate that non-activated microglia have an expedient and controlled release of EVs, whereas in an inflammatory condition microglia release a large variation of EVs that will perturb the normal homeostatic function of microglia. We also think that, upon LPS-stimulation, microglia respond to release populations of extracellular cargoes with a unique proteomic profile related to RNA transcription and translation. The existence of various RNA molecules in EVs is well-established including mRNA and microRNA. In fact, microRNAs can function as ligands for TLRs and induce immune responses or inhibit activation by suppressing TLR signaling [13, 47]. These RNAs are selectively sorted to EVs under different mechanisms [47]. However, the mechanisms responsible for this packaging are not clear. Thus, our study implies proteins potentially involved in such mechanisms that are increased when inflammation takes place. According to our proteomic data, we speculate that EVs could actually carry the whole machinery not only RNAs to recipient cells and surrounding tissue.

Finally, by comparing the protein cargoes from LPS-activated and non-activated BV2 microglia, we were able to identify potential candidates likely to be involved in the communication of microglial cells with other effector cells under inflammation. We found 49 proteins exclusively present in EVs in the presence of LPS. Among them, TUBB4B, HNRNPU, and SAA3 are proteins related to inflammation and neuropathology. Indeed, TUBB4B is a member of tubulin family and known as a component of cytoskeleton [48]. Liu X et al, suggested that TUBB4B may be a part of the same disease pathway as leucine-rich repeat kinase 2 (LRRK2), which is a crucial factor to understand the etiology of Parkinson’s disease (PD) [49]. HNRNPU acts as a key factor to maintain 3D structure of chromatin and has been reported as a posttranscriptional regulator for NF-κB inflammatory pathway [50]. In addition, SAA3 is a member of serum amyloid A (SAA) and acute phase protein accompanying with other inflammatory cytokines and chemokines [51]. It has been implied to play a role in the inflammatory processes occurring in Alzheimer’s disease (AD) and multiple sclerosis (MS) [51]. In addition to those proteins found exclusively in LPS EVs, we identified APOE in EVs from both LPS and control conditions. This protein is commonly present in membranes and is considered one of the most important lipoproteins involved in cholesterol shuttling between astrocytes and neurons along with being involved in remodeling and reorganization of neuronal networks after injury [34]. It is also one of the major genetic risk factors for late onset sporadic AD and can function as a ligand in receptor-mediated endocytosis with extracellular β-amyloid [34, 52].

## Conclusions

The present data show that upon activation by LPS, BV2 microglia release EVs with a distinct proteomic profile compared to control. Our data suggests that under these inflammatory conditions, MVs are the predominate EV, containing increased levels of TNF in particular, and to a lesser degree IL-6. We further provide evidence in vitro and in vivo that TNF signaling is important in quantitatively controlling EV release. Furthermore, through proteomic analysis, we are able to provide lists of proteins with the potential to modulate EV trafficking in microglia, in particular a change in EV proteins related to neuronal maintenance and protein translation after LPS activation. We believe that EV regulation in microglia and its specific role in neuroinflammation will be important to fully understand the inflammatory pathogenesis in neurodegenerative diseases.

## Additional files

Additional file 1: Supplementary figures for cytokines in conditioned medium from microglia show significant upregulations of TNF (*n* = 7), IL-1β (*n* = 7), IL5 (*n* = 7), and IL-6 (*n* = 3). Measured by multiplex ELISA (Unpaired *t* test, *P < 0.05; ***P < 0.001). (PDF 201 kb)

Additional file 2: Supplementary figures for cytokines in microglia-derived extracellular vesicles not altered after LPS treatment. Measured by multiplex ELISA (Unpaired *t* test, *P < 0.05; ***P < 0.001). (PDF 180 kb)

Additional file 3: Supplementary figures for the effect of TNF inhibition on dynamics of EV trafficking. Images were taken and then measured for fluorescent intensity. A) Representative images of BV2 cells cultured with PHK76-labeled EVs 12 h after different treatments, including pre-treatment of cells with either LPS (1 μg/ml) or etanercept (200 ng/ml) or in presence of both. Control (CTRL) was cells without any treatment. Cells without EV were regarded as baseline. Merged images of the indicated areas show PHK76 internalized cells (Scale bar, 50 μm). B) Comparison of total fluorescent intensity (IntDen) in BV2 cells after incubation of dye-labeled EVs. No significant differences were found between the conditions (one-way ANOVA, *n* = 3). (PDF 9799 kb)

Additional file 4: Supplementary figures for cytokines in serum from WT and TNF-KO mice before and 1 day after pMCAO. Measured by multiplex ELISA (one-way ANOVA followed by Tukey’s test for multiple comparisons, *n* = 3–6, **P < 0.01). (PDF 30 kb)

Additional file 5: Supplementary figures for quantification of extracellular vesicles in plasma from WT and TNF-KO mice subjected to pMCAO (Unpaired *t* test, *n* = 3). (PDF 18 kb)

## Abbreviations

AD: Alzheimer’s disease
APOE: Apolipoprotein E
BCA: Bicinchoninic acid assay
BSA: Bovine serum albumin
CNS: Central nervous system
DMEM: Dulbecco’s modified Eagle medium
ELISA: Enzyme-linked immunosorbent assay
EVs: Extracellular vesicles
FBS: Fetal bovine serum
FWHM: Full-width at half-maximum
GO: Gene Ontology
HNRNPU: Heterogeneous nuclear ribonucleoprotein U
HRP: Horse-radish protein
HSP8: Heat shock protein 8
IFNγ: Interferonγ
IL: Interleukin
iNOS: Inducible nitric oxide synthase
KC/GRO: C-X-C motif chemokine ligand 1
LC: Liquid chromatography
LPL: Lipoprotein lipase
LPS: Lipopolysaccharide
LRRK2: Leucine-rich repeat kinase 2
*m/z*: Mass to charge ratio
MS: Multiple sclerosis
MS/MS: Tandem mass spectrometry
MVB: Multivesicular endosomal compartments
MVs: Microvesicles
NLRP3: Nod-like receptor protein 3
NO: Nitric oxide
NTA: Nanoparticle tracking analysis
PBS: Phosphate-buffered saline
PD: Parkinson’s disease
PFA: Paraformaldehyde
pMCAO: Permanent middle cerebral artery occlusion
SAA3: Amyloid A-3 protein
solTNF: Soluble form of tumor necrosis factor
TEM: Transmission electron microscopy
TLR: Toll-like receptor
tmTNF: Transmembrane form of tumor necrosis factor
TNF: Tumor necrosis factor
TNF-KO: Tumor necrosis factor knockout
TUBB4B: Tubulin beta 4B
