# Supplementary material for: Inflammation leads to distinct populations of extracellular vesicles from microglia
Source: J Neuroinflammation. 2018 May 28;15:168. doi: 10.1186/s12974-018-1204-7 (PMC5972400; doi:10.1186/s12974-018-1204-7)
Supplement: Supplementary file 1 — Supplementary figures for cytokines in conditioned medium from microglia show significant upregulations of TNF (n = 7), IL-1β (n = 7), IL5 (n = 7), and IL-6 (n = 3). Measured by multiplex ELISA (Unpaired t test, *P < 0.05; ***P < 0.001). (PDF 201 kb) [file 12974_2018_1204_MOESM1_ESM.pdf]

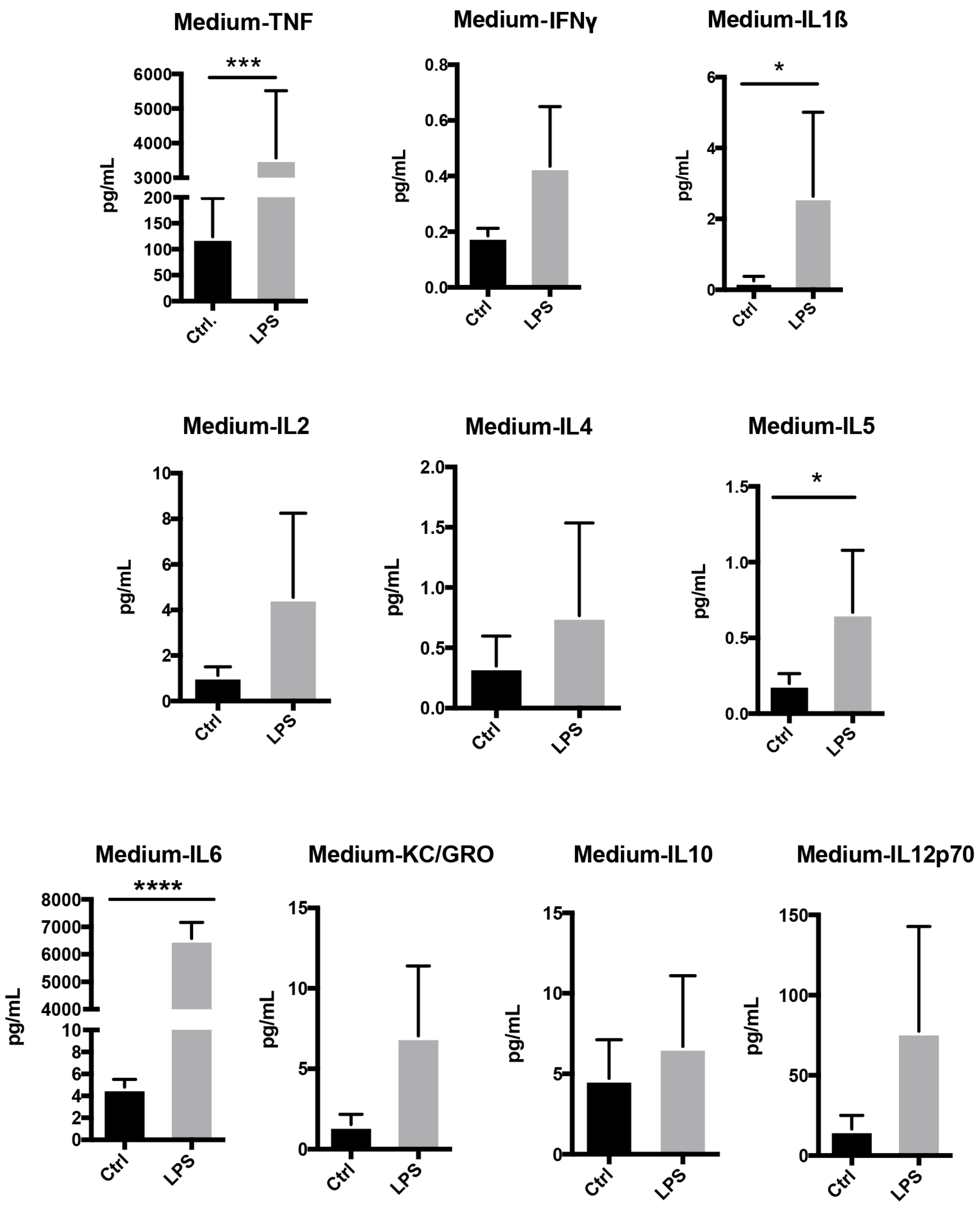
Medium-TNF
***
6000
5000
4000
3000
200
150
100
50
0
pg/mL
Ctrl.
LPS
Medium-IFNγ
0.8
0.6
0.4
0.2
0.0
pg/mL
Ctrl
LPS
Medium-IL1ß
*
6
4
2
0
pg/mL
Ctrl
LPS
Medium-IL2
10
8
6
4
2
0
pg/mL
Ctrl
LPS
Medium-IL4
2.0
1.5
1.0
0.5
0.0
pg/mL
Ctrl
LPS
Medium-IL5
*
1.5
1.0
0.5
0.0
pg/mL
Ctrl
LPS
Medium-IL6
****
8000
7000
6000
5000
4000
10
8
6
4
2
0
pg/mL
Ctrl
LPS
Medium-KC/GRO
15
10
5
0
pg/mL
Ctrl
LPS
Medium-IL10
15
10
5
0
pg/mL
Ctrl
LPS
Medium-IL12p70
150
100
50
0
pg/mL
Ctrl
LPS
