# Supplementary figures and images for: Inflammation leads to distinct populations of extracellular vesicles from microglia
Source: J Neuroinflammation. 2018 May 28;15:168. doi: 10.1186/s12974-018-1204-7 (PMC5972400; doi:10.1186/s12974-018-1204-7)

**EVs-IFN $\gamma$**

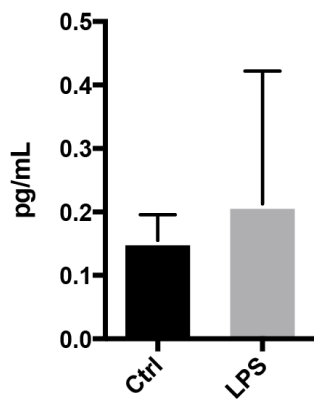

**EVs-IL1 $\beta$**

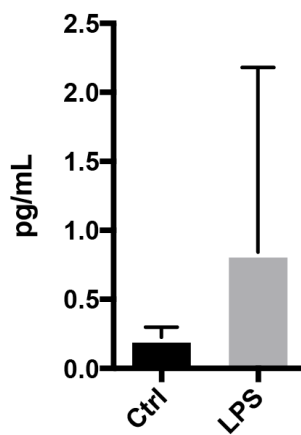

**EVs-IL2**

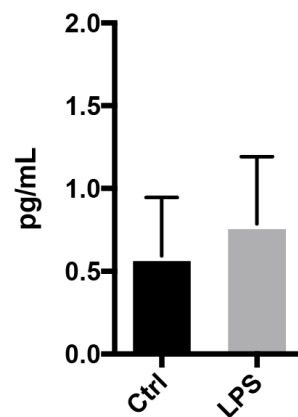

**EVs-IL4**

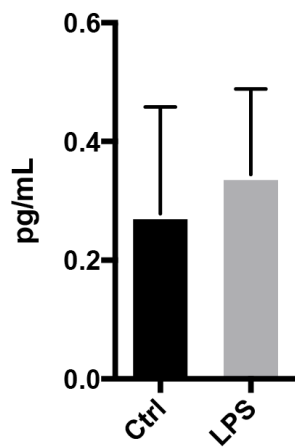

**EVs-IL5**

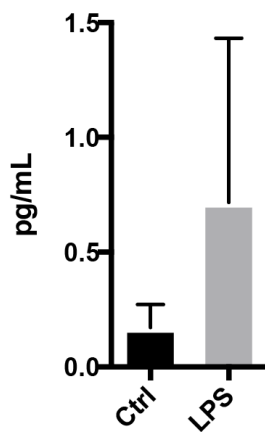

**EVs-KC/GRO**

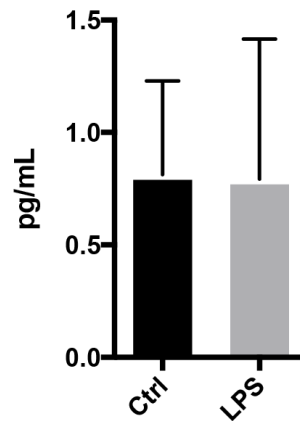

**EVs-IL10**

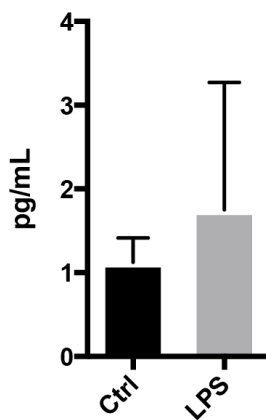

**EVs-IL12p70**

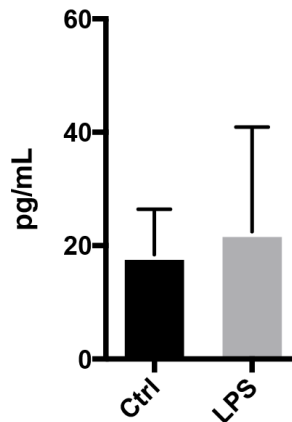

Supplement: Supplementary file 2 — Supplementary figures for cytokines in microglia-derived extracellular vesicles not altered after LPS treatment. Measured by multiplex ELISA (Unpaired t test, *P < 0.05; ***P < 0.001). (PDF 180 kb) [file 12974_2018_1204_MOESM2_ESM.pdf]

**A****PHK76-EV****Bright Field / PHK76****Enlarged****Cells + No EV**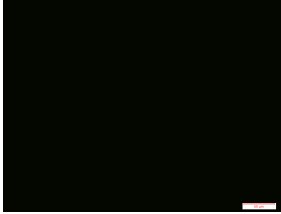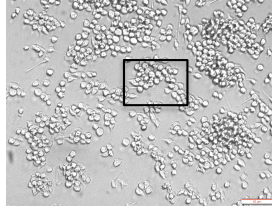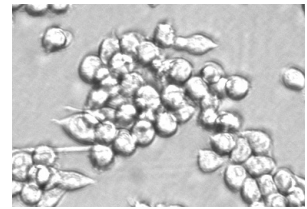**CTRL + EV**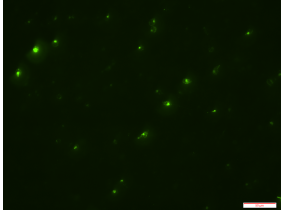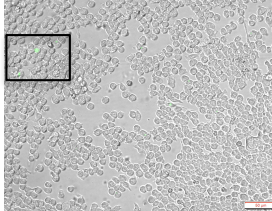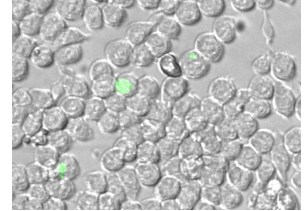**LPS + EV**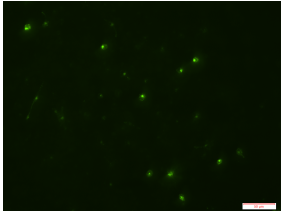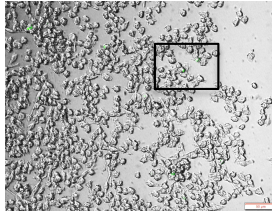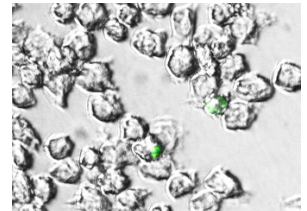**LPS  
+  
Etanercept  
+  
EV**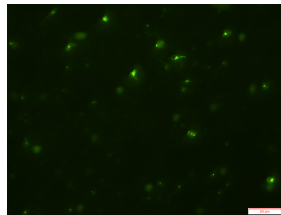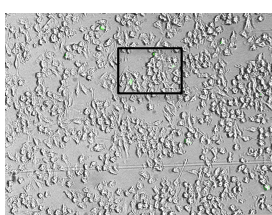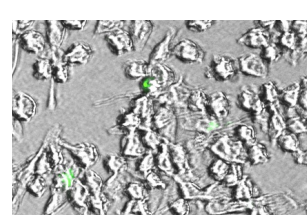**Etanercept + EV**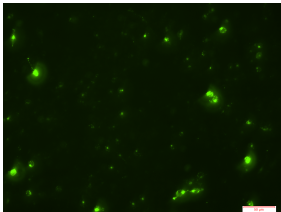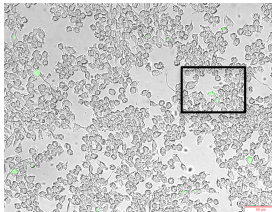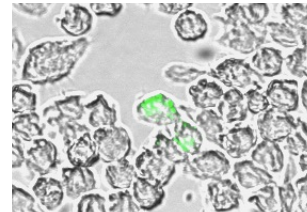**B**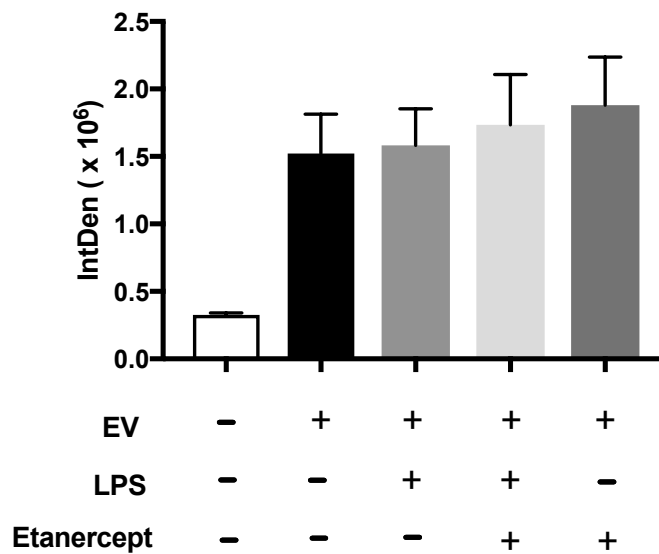

Supplement: Supplementary file 3 — Supplementary figures for the effect of TNF inhibition on dynamics of EV trafficking. Images were taken and then measured for fluorescent intensity. A) Representative images of BV2 cells cultured with PHK76-labeled EVs 12 h after different treatments, including pre-treatment of cells with either LPS (1 μg/ml) or etanercept (200 ng/ml) or in presence of both. Control (CTRL) was cells without any treatment. Cells without EV were regarded as baseline. Merged images of the indicated areas show PHK76 internalized cells (Scale bar, 50 μm). B) Comparison of total fluorescent intensity (IntDen) in BV2 cells after incubation of dye-labeled EVs. No significant differences were found between the conditions (one-way ANOVA, n = 3). (PDF 9799 kb) [file 12974_2018_1204_MOESM3_ESM.pdf]

**TNF**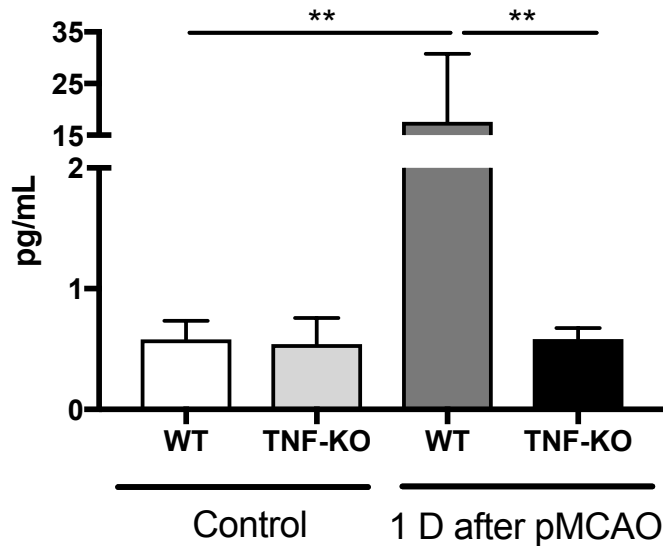**IL-2**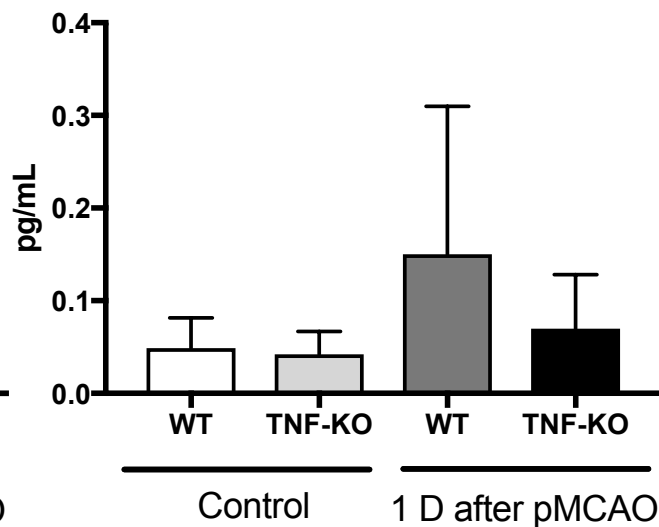**IFN $\gamma$** 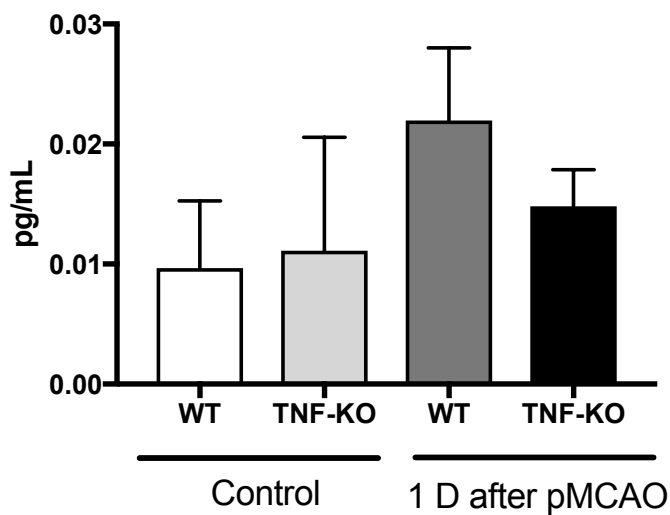**IL-4**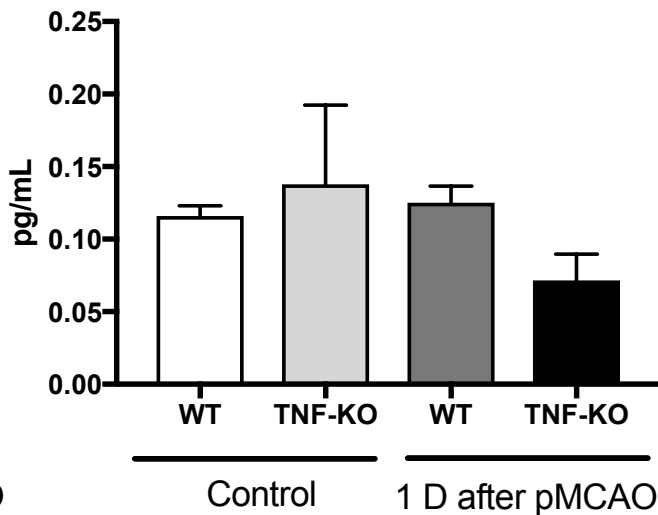

Supplement: Supplementary file 4 — Supplementary figures for cytokines in serum from WT and TNF-KO mice before and 1 day after pMCAO. Measured by multiplex ELISA (one-way ANOVA followed by Tukey’s test for multiple comparisons, n = 3–6, **P < 0.01). (PDF 30 kb) [file 12974_2018_1204_MOESM4_ESM.pdf]

# 1 D after pMCAO

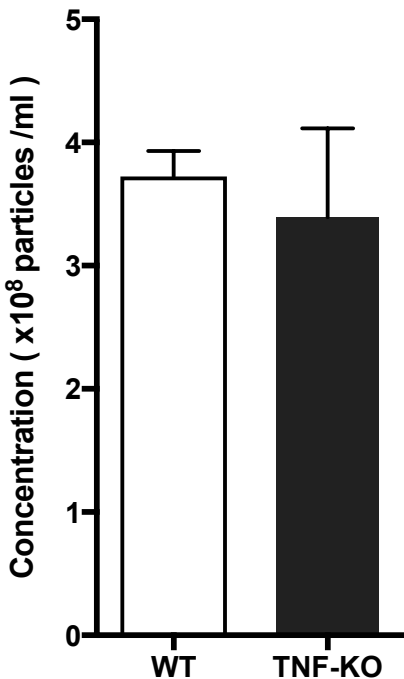

Supplement: Supplementary file 5 — Supplementary figures for quantification of extracellular vesicles in plasma from WT and TNF-KO mice subjected to pMCAO (Unpaired t test, n = 3). (PDF 18 kb) [file 12974_2018_1204_MOESM5_ESM.pdf]
